# Asystematic review and meta-analysis of clinical prognostic factors linked to extravesical recurrence after radical nephroureterectomy to treat upper tract urothelial carcinoma

**DOI:** 10.3389/fonc.2024.1475044

**Published:** 2024-11-26

**Authors:** Guanlan Zhang, Zhaoqiang Jiang, Jiawei Chen, Ying Zhao, Jianan Wang, Jinxing Liu, Zhenshan Ding, Lei Shan

**Affiliations:** ^1^ Urology Department, Henan Provincial People’s Hospital, Zhengzhou, China; ^2^ Department of Medicine, Graduate School, Henan University, Kaifeng, China; ^3^ Beijing Advanced Innovation Center for Soft Matter Science and Engineering, College of Life Science and Technology, Beijing University of Chemical Technology, Beijing, China; ^4^ Urology Department, China-Japan Friendship Hospital, Beijing, China

**Keywords:** urinary tract, urothelial carcinoma, ureter, extravesical recurrence, predictor, meta-analysis

## Abstract

**Objective:**

Numerous studies have investigated predictors of intravesical recurrence following radical nephrectomy (RNU) in patients with upper urinary tract uroepithelial carcinoma (UTUC). In contrast, extravesical recurrence (EUR) has received less focus. Consequently, this study aims to evaluate the significant predictors of EUR after RNU through a systematic review of the literature and a meta-analysis.

**Methodology:**

We conducted a computerized bibliographic search across PubMed, Embase, and Cochrane databases to identify reports that include detailed results from multivariate analyses of predictors of EUR. Adhering to the Preferred Reporting Items for Systematic Reviews and Meta-Analyses (PRISMA) guidelines and the AMSTAR (Assessing the Methodological Quality of Systematic Reviews) criteria, we selected thirteen retrospective studies, each with a sample size exceeding 100 cases. Using Review Manager 5.4 software, we performed cumulative analyses of available HR and their corresponding 95% confidence intervals to evaluate potential predictors of EUR.

**Results:**

Our findings indicate that patient-specific predictors include preoperative Ki-67 with a HR of 3.61 (P = 0.003), neutrophil-to-lymphocyte ratio with an HR of 2.20 (P = 0.0005), and glomerular filtration rate with an HR of 3.35 (P = 0.0009). Tumor-specific predictors identified were tumor stage with an HR of 4.67 (P < 0.00001), lymphovascular invasion with an HR of 2.37 (P = 0.004), and lymph node status with an HR of 2.68 (P < 0.0001). Regarding treatment-specific predictors, positive surgical margins were associated with an HR of 3.97 (P = 0.0005), and adjuvant chemotherapy was associated with an HR of 1.65 (P = 0.03).

**Discussion:**

Our study identified three significant predictors across patient, tumor, and treatment dimensions for extravesical recurrence following radical nephroureterectomy in patients with upper urinary tract uroepithelial carcinoma. We hypothesize that history of bladder cancer, platelet-to-lymphocyte ratio, and urinary cytology could also be strong predictors of post- RNU extravesical recurrence in patients with upper UTUC, assuming adequate sample size and controlled heterogeneity. This research aims to provide urological clinicians with enhanced guidance for postoperative decision-making.

## 1 Introduction

Malignant tumors originating from the uroepithelium of the renal pelvis, calices, and ureters are collectively termed UTUC. This group represents 5-10% of all uroepithelial carcinomas (1), making it a relatively rare condition. In Western countries, the incidence is approximately 12 cases per 100,000 individuals (2, 3). In Asian populations, the prevalence of UTUC has been rising in recent years due to advancements in diagnostic techniques, such as radiology and endoscopy. The five-year survival rate ranges from 30% to 70%, predominantly influenced by tumor size, stage, grading, and recurrence frequency. Although the etiology of UTUC remains unclear, smoking, herbal medicines, chronic infections, and occupational exposure to carcinogens are established risk factors (4). The current clinical standard for treating UTUC involves radical nephroureterectomy and ureteral resection, combined with sleeve cystectomy (RUN) (5).

Previous research has predominantly focused on intravesical recurrence post- RNU for upper UTUC, largely because the probability of intravesical recurrence ranges from 15% to 50% (6, 7). Beyond intravesical sites, extra-urinary recurrence, particularly extra-urethral, occurs in over 20% of patients and carries significant prognostic consequences (8). Although numerous studies have investigated clinical factors associated with postoperative recurrence in UTUC, less attention has been given to extra-urinary recurrence. Furthermore, the pathogenesis of intravesical recurrence post-RNU remains poorly understood (9), and there is no international consensus on factors influencing extravesical recurrence in UTUC patients. Recent years have seen some reduction in local recurrence or distant metastasis through adjuvant radiotherapy, though the overall benefits remain limited (10). Identifying high-risk groups for postoperative extravesical bladder recurrence, targeting treatment to mitigate this risk, and enhancing five- and ten-year survival rates for UTUC patients are not only clinically challenging but also critical for improving prognosis (11). Accordingly, we reviewed recent literature and proposed an investigation into clinical and prognostic factors associated with extra-urinary recurrence in UTUC. Our objective is to assess the significant predictors for extravesical recurrence by analyzing a substantial cohort of patients who underwent RNU for UTUC through systematic literature review and meta-analysis.

## 2 Materials and methods

### 2.1 Literature search and article selection

In March 2024, a comprehensive search of electronic databases, including PubMed, Embase, and the Cochrane Library, was conducted by two authors (GL Z. and ZQ J.). They employed a free-word search strategy. The search terms used were: “Extraurothelial recurrence” AND “Nephroureterectomy” AND (“Upper tract” OR “Upper urinary tract” OR “Renal pelvis” OR “Ureter”) AND (“Urothelial carcinoma” OR “Renal pelvis” OR “Carcinoma” OR “Transitional cell carcinoma”). The protocol was limited to documents concerning only “Humans,” published in “English,” and within the “last ten years.”

In accordance with the PRISMA guidelines, our study eligibility was determined using the Population, Intervention, Comparator, Outcome, and Study design (PICOS) framework (12). Studies were included in this systematic review and meta-analysis if they involved patients diagnosed with cancer post- RNU for UTUC (P), compared these patients to those without cancer (C), aimed to identify independent clinicopathologic predictors of EUR (O), and utilized multivariate logistic regression analysis (S). Exclusions applied to case reports, meeting abstracts, letters, editorials, and review articles throughout the review process.

**Figure 1:**
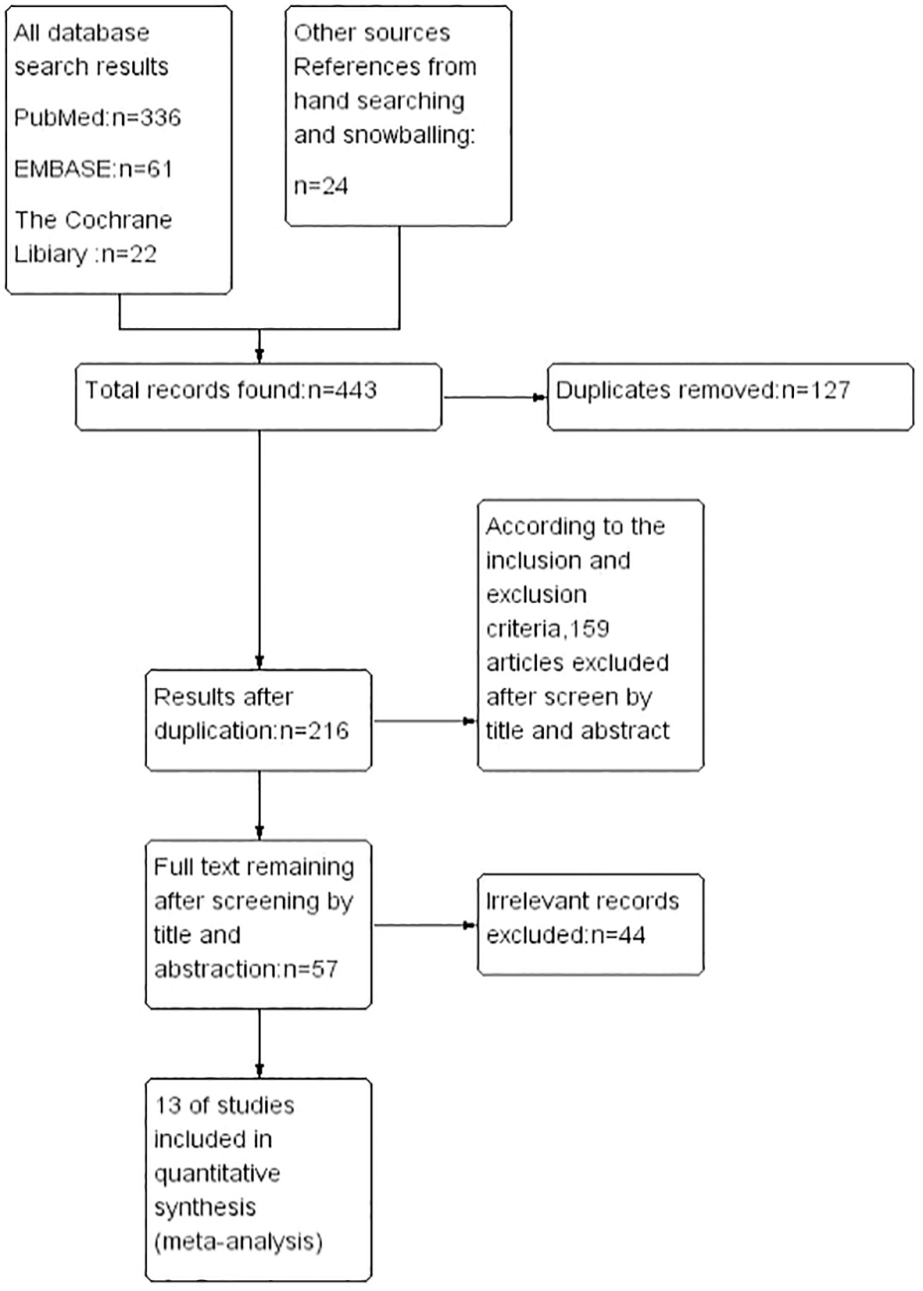
PRISMA flowchart.

Studies were selected according to the following criteria:

1. Only large studies that included more than 100 patients who had been treated exclusively with RNU.
2. Only studies that defined EUR as a pathologically confirmed occurrence of cancer after RNU.
3. Only studies that excluded patients with previous cancer or tumor of systemic, or that used previous cancer or tumor of systemic as a variable for adjustment in multivariate analysis.
4. Only studies that provided hazard ratios (HRs) from multivariate logistic regression analyses with their corresponding 95% confidence intervals (CIs).Following the removal of duplicates, two authors (GL Z. and ZQ J.) independently reviewed 443 abstracts, culminating in the selection of 89 studies for detailed full-text evaluation. Discrepancies regarding study inclusion were resolved through consultation with the senior author (Z G), who oversaw the systematic review process. Adhering to the established inclusion criteria, a final selection of 13 articles, published between 2014 and 2023, was made (13–25). The systematic literature search and selection process is illustrated in the PRISMA flow chart (**Figure 1**). It is essential to recognize that this study adheres to the PRISMA (Preferred Reporting Items for Systematic Reviews and Meta-Analyses) (26) and AMSTAR (Assessing the Methodological Quality of Systematic Reviews) guidelines (27), we present the overall characteristics of the included literature in a tabular form as shown in (**Table 1**).

**Table 1:** Overall characteristics of extravesical recurrence after radical nephroureterectomy to treat upper tract urothelial carcinoma.

Studies	No. of Patients	No. of EUR(%)	Median time to EUR, mo [range]	Median follow-up, mo [range]
Carrion et al. (2016) (13)	117	36(30.8)	11.4[1.0-46.0]	20.0[3.0-97.0]
Dzamic et al. (2015) (14)	238	65(27.3)	17.6[3.0-73.0]	34.5[1.0-154.0]
Ito et al. (2014) (15)	135	21(15.6)	8.4[0.6-49.3]	29.2[1.0-157.0]
Ito et al. (2016) (16)	150	12(8.0)	6.5[1.0-13.0]	50.3[1.0-160.0]
Kawamura et al. (2021) (17)	135	44(33.0)	NR	53.6[0.4-240.5]
Kawamura et al. (2023) (18)	211	50(23.7)	NR	65.0[1.4-249.0]
Kuroda et al. (2019) (19)	187	32(16.0)	NR	49.2[3.4-209.2]
Kuroda et al. (2021) (20)	145	41(28.3)	NR	54.2[3.4-209.2]
Luo et al. (2023) (21)	521	130(25.0)	NR	40.0[19.0-70.0]
Luo et al. (2023) (22)	220	61(27.7)	10.4[1.0-62.0]	42.0[2.0-143.0]
Milojevic et al. (2015) (23)	238	65(27.3)	17.6[3.0-73.0]	34.5[1.0-154.0]
Sawazaki et al. (2016) (24)	114	21(18.4)	10.4[3.0-60.0]	58.0[4.0-129.0]
Sevillano et al. (2017) (25)	102	57(55.9)	29.8[1.0-44.0]	46.0[6.0-103.0]
**Pooled results**	**2513**	**635(25.3)**	**10.9[1.0-54.7]**	**46.0[2.0-154.0]**

EUR, extravesical recurrence; NR, not reported. The results of the merger have been bolded.

### 2.2 Assessment of study quality

The 13 studies included in this review were characterized by their retrospective design. Despite this limitation, each was deemed to be of high methodological quality based on the Newcastle-Ottawa Scale, which is endorsed by the Cochrane Collaboration (28). This scale is designed to evaluate the quality of nonrandomized studies and facilitates the integration of quality assessments into the interpretation of meta-analytic outcomes. Employing a star system that assigns a score from 0 to 9, each study was independently evaluated by two authors (GL Z. and ZQ J.) based on predefined inclusion criteria, group comparability, and the ascertainment of relevant outcomes.

### 2.3 Data extraction

Data from all included studies were independently extracted by two authors (GL Z. and ZQ J.) and then crosschecked to verify accuracy. Discrepancies in data extraction were resolved through consultation with the senior author (MRZ G.). Initially, the number of patients, along with their clinicopathologic features, the prevalence of EUR, the median time to EUR occurrence, and the median follow-up period for assessing the overall characteristics of cancer post- UTUC were recorded. Subsequently, HRs for potential predictors of EUR, included in multivariate models, were extracted along with their corresponding 95% CIs to facilitate cumulative analyses.

### 2.4 Outcome measures

The primary outcome assessed the adjusted effect on EUR in relation to patient-specific, tumor-specific, and treatment-specific factors. Patient-specific predictors analyzed included age, gender, history of bladder cancer (BCa), and hydronephrosis. Tumor-specific predictors encompassed preoperative urinary cytology, tumor location, size, pT stage, pN stage, grade, presence of concomitant CIs, lymphovascular invasion, and necrosis. Treatment-specific predictors evaluated were the surgical approach, management of the distal ureter, margin status, and adjuvant chemotherapy.

### 2.5 Statistical analyses

A meta-analysis was conducted for each potential predictor of EUR using the predictor effect (PE) and its standard error (sePE), derived from the available adjusted HRs and their corresponding 95% CIs. The cumulative effects of the factors of interest were assessed using the inverse variance method. Depending on the between-study heterogeneity, either a fixed-effect or random-effect model was applied. Statistical heterogeneity was evaluated using both the Cochran Q test and the I² statistic, the latter quantifying the proportion of total variation across studies attributable to heterogeneity rather than chance. A p-value less than 0.05 for the Cochran Q test or an I² statistic exceeding 50% was indicative of significant heterogeneity among the studies. The potential for small-study effects and publication bias was investigated through visual inspection of funnel plots for all comparisons (29). The meta-analysis of comparable data was executed using Review Manager 5.4 software.

## 3 Results

### 3.1 Patient-specific predictors of EUR

#### 3.1.1 Age

Of all the 13 articles included in this study, only the article by Dzamic (14) discussed the relationship between the influence of the age factor and extravesical recurrence, and in this article, with a sample size of 238, the mean age of the included patients was 66.5 years, with 65 extravesical recurrences occurring across the entire age range. Age-related EUR risk-corrected HRs and their 95% confidence intervals were also provided (HR 1.42, 95%CI 0.71–2.82; p = 0.32). However, because the other included articles didn’t give a risk correction index for the age factor for extravesical recurrence, they were not included in the outcome analysis, The amount of data was too small for us to consider age as a significant predictor of EUR occurrence.

#### 3.1.2 Sex

Of all the 13 articles included in this study, only the article by Dzamic (12) discussed the influence of gender factors in relation to extravesical recurrence, with 55.4% of men and 44.6% of women out of a study population of 238, clearly there were more male than female patients, while at the same time, extravesical recurrences occurred in more male patients (58.5%) than the rate of extravesical recurrences in female patients (41.5%), and the article also gives the gender-related EUR risk-corrected HR and its 95% confidence interval(HR 0.83, 95%CI 0.49–1.39; p = 0.49). However, because relevant data were not available in the other included articles, they were not included in the outcome analyses, nor can we consider gender as a significant predictor of the occurrence of EUR,.

#### 3.1.3 Previous bladder cancer

The Dzamic 2015 (14) and Luo 2023 (21) articles provide EUR risk-corrected HRs and their 95% confidence intervals associated with prior bladder cancer history, As I^2^ = 83% > 50%, there was significant heterogeneity in the data, and a cumulative analysis of available HR using a random effects model showed that, the HR for both groups was 1.72, 95%CI0.50-5.94;overall effect P=0.39>0.05,therefore,we concluded that prior BCa was not a significant predictor of EUR in the included studies.

#### 3.1.4 Preoperative hydronephrosis

Only two studies (20, 22) evaluated preoperative hydronephrosis as a factor for EUR after RNU. Of the 365 patients included, 147 (40.27%) had preoperative hydronephrosis with a risk-correction index and 95% confidence interval of(HR1.438, 95%CI0.685-3.240;p=0.345), (HR1.416, 95%CI0.844-2.374;p=0.188).However, there was no heterogeneity in the observed outcomes according to the 0% I^2^ statistic (p = 0.97) using a fixed effects model (HR1.42, 95%CI0.93-2.19, overall effect P=0.11>0.05), There was no significant difference and it was concluded that preoperative hydronephrosis was not a significant predictor of EUR.

#### 3.1.5 Preoperative anemia

Three studies (14, 23, 25) analyzed preoperative patient anemia as a factor for EUR after RNU. There are conflicting results regarding the risk predictive value of preoperative anemia and EUR, with both authors, Dzamic and Sevillano, concluding that there is no significant difference between anemia and the predictors of EUR, whereas Milojevic’s study demonstrated that anemia predicted EUR with a significance of P=0.01.Based on the statistic of I^2^ = 47%, the observed heterogeneity of outcomes was small, and a meta-analysis of available HRs using a fixed-effects model showed that Preoperative anemia(HR 1.40, 95% CI 0.98-2.00; p = 0.06>0.05)not considered a significant predictor of EUR.

#### 3.1.6 Proliferator-associated nuclear antigen Ki-67

Three studies (21, 22, 24) provided risk-corrected HRs associated with proliferator-associated nuclear antigen Ki-67 and EUR after RNU, all three studies considered Ki-67 to be significantly associated with the predicted risk of EUR, with p-values of p = 0.03, p < 0.001, and p = 0.032, respectively, and based on the statistic of I2 = 74% (p = 0.02), it was considered that there was observed outcome Heterogeneity, using a random effects model, a meta-analysis of available HRs showed that Ki-67 (HR 3.61, 95% CI 1.54-8.44; overall effect p = 0.003 < 0.05) was considered a significant predictor of EUR

#### 3.1.7 Neutrophil to lymphocyte ratio

Three studies (15, 19, 22) analyzed the preoperative neutrophil count to lymphocyte count ratio as a factor for EUR, and there were conflicting results regarding the risk predictive value of preoperative NLR and EUR, with both authors Ito and Kuroda concluding that anemia was not significantly different from the predictors of EUR, whereas the study by Luo2023 demonstrated that the significance of preoperative NLR in predicting EUR was p = 0.003. Based on the statistic of I^2^ = 0% (p = 0.74), the data were homogeneous and a fixed effects model could be used, and a meta-analysis of the available HRs showed that NLR (HR 2.20, 95% CI 1.42-3.43; overall effect p = 0.0005) was considered a significant predictor of EUR.

#### 3.1.8 eGFR

**Figure 2:**
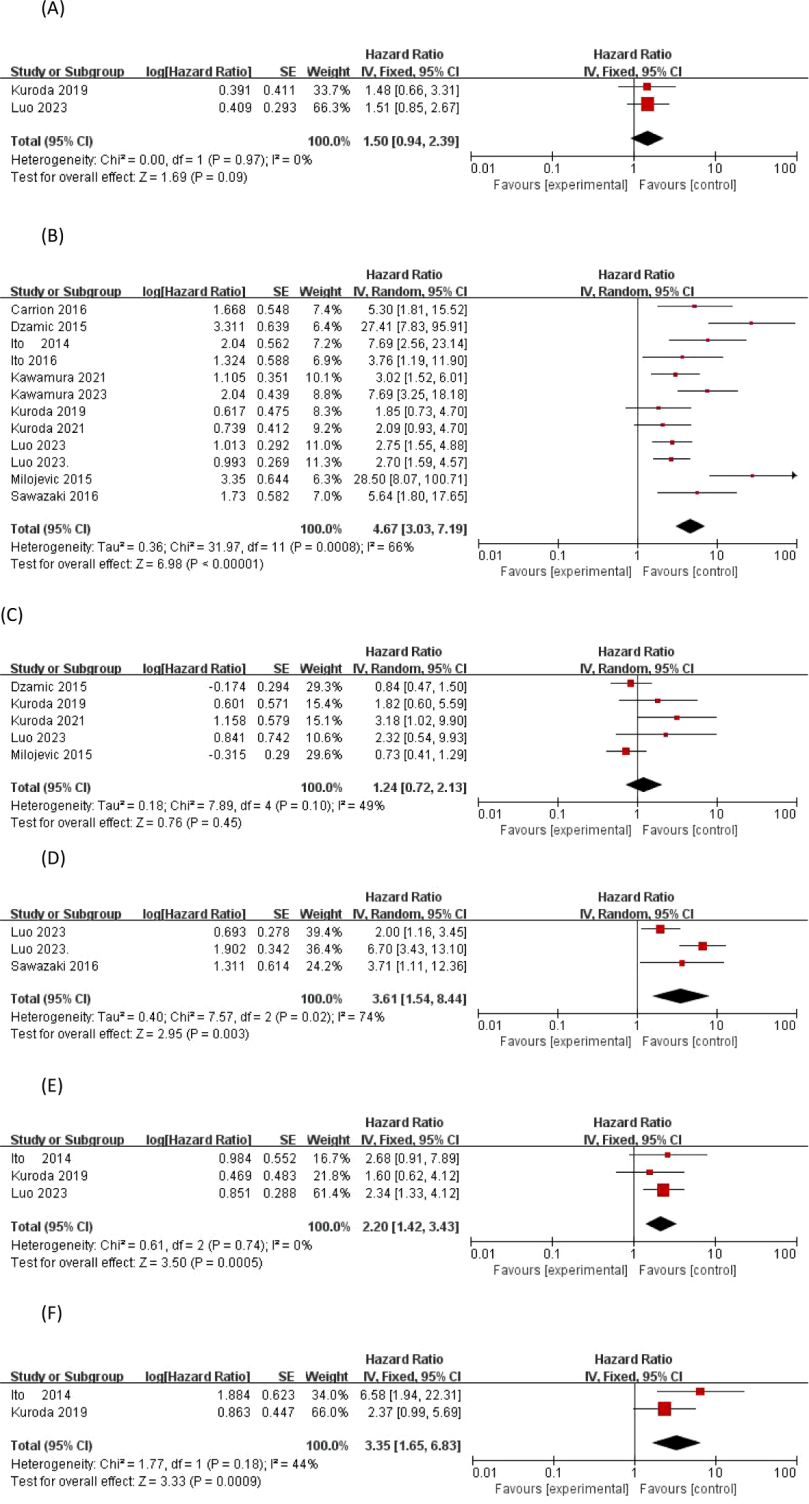
Forrest plots of meta-analyses of patient-specific predictors of EUR: **(A)** previous bladder cancer, **(B)** Preoperative hydronephrosis, **(C)** Preoperative anemia, **(D)** Proliferator-associated nuclear antigen Ki-67, **(E)** Neutrophil to lymphocyte ratio and **(F)** eGFR. EUR, extravesical recurrence.

Two articles provided risk-corrected HRs associated with glomerular filtration rate and EUR, and there were conflicting results regarding the risk-predictive value of preoperative eGFR and EUR, with Ito’s study (15) demonstrating that preoperative eGFR significantly predicted EUR after RNU, with a p-value of 0.0026, and Kuroda’s study (19) to the contrary. Based on the statistic of I^2^ = 44% (p = 0.18), the heterogeneity of outcomes was observed to be low, and a meta-analysis of the available HRs using a fixed-effects model showed that eGFR (HR 3.35, 95% CI 1.65-6.83; overall effect p = 0.0009 < 0.05) was considered to be a significant predictor of EUR, as shown in **Figure 2**.

### 3.2 Tumor-specific predictors of EUR

#### 3.2.1 Preoperative urinary cytology

Abnormal uroepithelial cells on preoperative cytological examination were found in the urine of 156 (42.73%) patients included in two studies (n = 365). The risk-corrected indices and 95% confidence intervals for the two studies were (19, 22) (HR 1.479, 95% CI 0.679-3.398; p = 0.330), (HR 1.505, 95% CI 0.848-2.671; p = 0.163), respectively. The data were homogeneous and a cumulative analysis of the available HRs using a fixed-effects model showed a (HR of 1.50, 95% CI 0.94-2.39; the overall effect size was p=0.09>0.05),so it can be assumed that a positive preoperative urinary cytology test is not a significant predictor of EUR.

#### 3.2.2 Tumor location

Only the article by Dzamic (14) discussed the relationship between the effect of tumor location (including renal pelvic and ureteral tumors) and extravesical recurrence and provided a risk-corrected HR for tumor location-associated EUR and its 95% confidence interval (HR 1.21, 95% CI 0.66-2.23; p = 0.54), but as the other included articles did not give a tumor location risk-corrected index for extravesical recurrence, we didn’t consider tumor location to be a significant predictor of EUR.

#### 3.2.3 Tumor focality

Only the article by Dzamic (14) discussed the relationship between the effect of tumor multifocality (both single and multiple) and extravesical recurrence and provided a risk-corrected HR for tumor multifocality-associated EUR and its 95% confidence interval (HR 1.1, 95% CI 0.63-2.29; p = 0.58), but since the other included articles did not give a risk-corrected HR for tumor multifocality on the risk-corrected index for extravesical recurrence, we did not consider tumor multifocality to be a significant predictor of EUR.

#### 3.2.4 Tumor size

One study(n = 238) (14) reported median primary tumor size as a factor for EUR, with a range encompassing ≤3 cm and >3 cm, with an HR of 0.94 and a 95% confidence interval of 0.53-1.65, P = 0.83 > 0.05. As the other included articles did not give a risk-corrected index of tumor size for extravesical recurrences, we do not consider tumor size to be a significant predictor of EUR.

#### 3.2.5 Tumor stage

Of the 2411 patients included in the 12 studies providing risk-corrected HRs associated with EUR tumor staging, a total of nine studies showed significant differences between invasive and superficial UTUC, with their risk-corrected indices and 95% confidence intervals being Dzamic 2015 (14) (HR27.4, 95%CI 7.83–95.8;P<0.001), Luo2023 (22) (HR2.755, 95%CI 1.554–4.886;P<0.001), Milojevic 2015 (23) (HR28.5, 95%CI 8.08–100.9;P=0.001), Carrion 2016 (13) (HR5.3, 95%CI 1.8–15.4;P=0.028), Ito 2014 (15) (HR7.692, 95%CI 2.564–23.256;P=0.0003), Ito 2016 (16) (HR3.759, 95%CI1.188–11.905;P=0.0244), Kawamura 2021 (17) (HR3.02, 95%CI 1.52–6.02;P=0.002), Kawamura 2023 (18) (HR7.69, 95%CI 3.25–18.18;P<0.0001), Sawazaki 2016 (24) (HR5.64, 95%CI 1.79–17.5;P=0.03).I2 = 66% > 50%, a random-effects model was chosen, and a cumulative analysis of the available HRs showed an HR of 4.67 with a 95% CI of 3.03-7.19; the overall effect size Z-value = 6.98 with a P < 0.00001, so we concluded that invasive pT staging was a significant predictor of EUR.

#### 3.2.6 Tumor grade

A total of 5 out of 13 included articles provided a corrected HR for tumor grade-related EUR risk and its 95% confidence interval, but a significant difference between high-grade and low-grade UTUC and EUR was only found in the data from the study of Kuroda 2021 (20), with an HR of 3.182, 95% CI 1.162-11.223; p = 0.023 < 0.05. According to the I^2^ statistic of 49% (p = 0.10), less heterogeneity in outcomes between studies was observed, and a meta-analysis of the available HRs using a fixed-effects model showed that tumor grade (HR = 1.24, 95% CI 0.72-2.13; p = 0.45 > 0.05) was not a significant predictor of EUR.

#### 3.2.7 Tumor histology

Two studies published by Kuroda in 2019 and 2021 (19, 20) reported tumor histology as a factor for EUR, including UC alone or with other components, which were not significantly different, and the data were suggestive of homogeneity, and a meta-analysis of the available HRs using a fixed-effects model showed that tumor grade (HR=1.81, 95% CI01.00-3.28; P=0.05) was not a significant predictor of EUR.

#### 3.2.8 Concomitant carcinoma in situ

Three studies reported concomitant CIS as a factor in EUR, and although two studies, Kawamura 2021 (17) and Kawamura 2023 (18), demonstrated that concomitant CIS was significantly associated with the occurrence of BCa after prior UTUC with p-values of 0.021, and <0.0001, respectively, there was heterogeneity in the observed outcomes according to the 70% I^2^ statistic (p = 0.04). A meta-analysis of available HRs showed that concomitant CIS (HR 1.96, 95% CI 0.99-3.85; p = 0.05) was not a significant predictor of EUR.

#### 3.2.9 Lymphovascular invasion

Eight studies (14, 17–23) provided a corrected HR for the risk associated with LVI for EUR, and there were conflicting results regarding the predictive value of LVI for the risk of EUR, with six studies dominating the opinion that LVI has a significant predictive value for EUR. At the same time, according to the I^2^ statistic 86% (p < 0.00001), this translates into inter-study heterogeneity of observations. A meta-analysis of available HRs showed that LVI (HR 2.37, 95% CI 1.33-4.22; p = 0.004 < 0.05) was a significant predictor of EUR.

#### 3.2.10 Lymph node status

**Figure 3:**
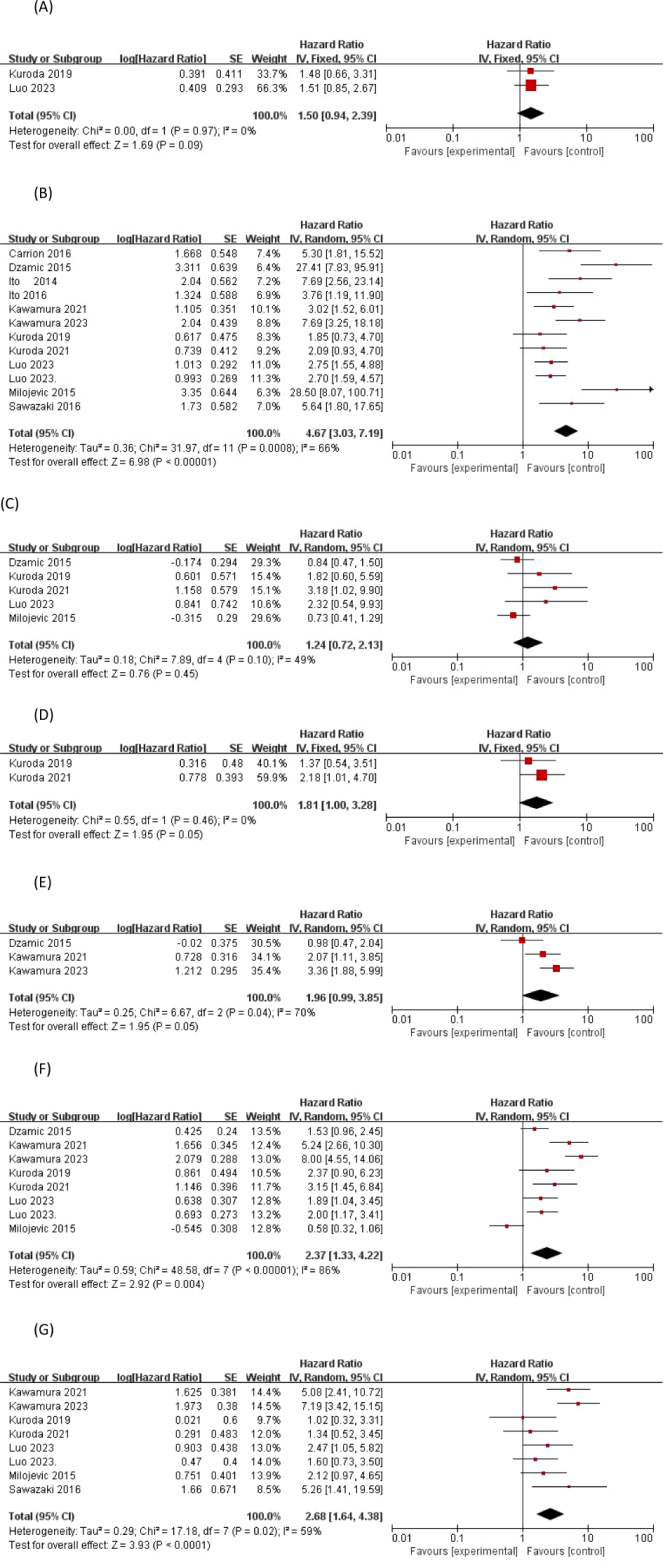
Forrest plots of meta-analyses of tumor-specific predictors of EUR: **(A)** Preoperative urinary cytology, **(B)** Tumor stage, **(C)** Tumor grade, **(D)** Tumor histology, **(E)** Concomitant carcinoma *in situ*, **(F)** Lymphovascular-invasion, and **(G)** Lymph node status. EUR, extravesical recurrence.

A total of eight studies (17–24) provided corrected HRs for LNS and EUR-related risk, and there were conflicting results regarding the predictive value of LNS for EUR risk. Based on the statistic of I2 = 59% (p = 0.02), there was heterogeneity of studies observing the results, and a meta-analysis of the available HRs showed that LNS (HR 2.68, 95% CI 1.64-4.38; overall effect p < 0.0001) could be considered a significant predictor of EUR, as shown in **Figure 3**.

### 3.3 Treatment-specific predictors of EUR

#### 3.3.1 Positive surgical margins

**Figure 4:**
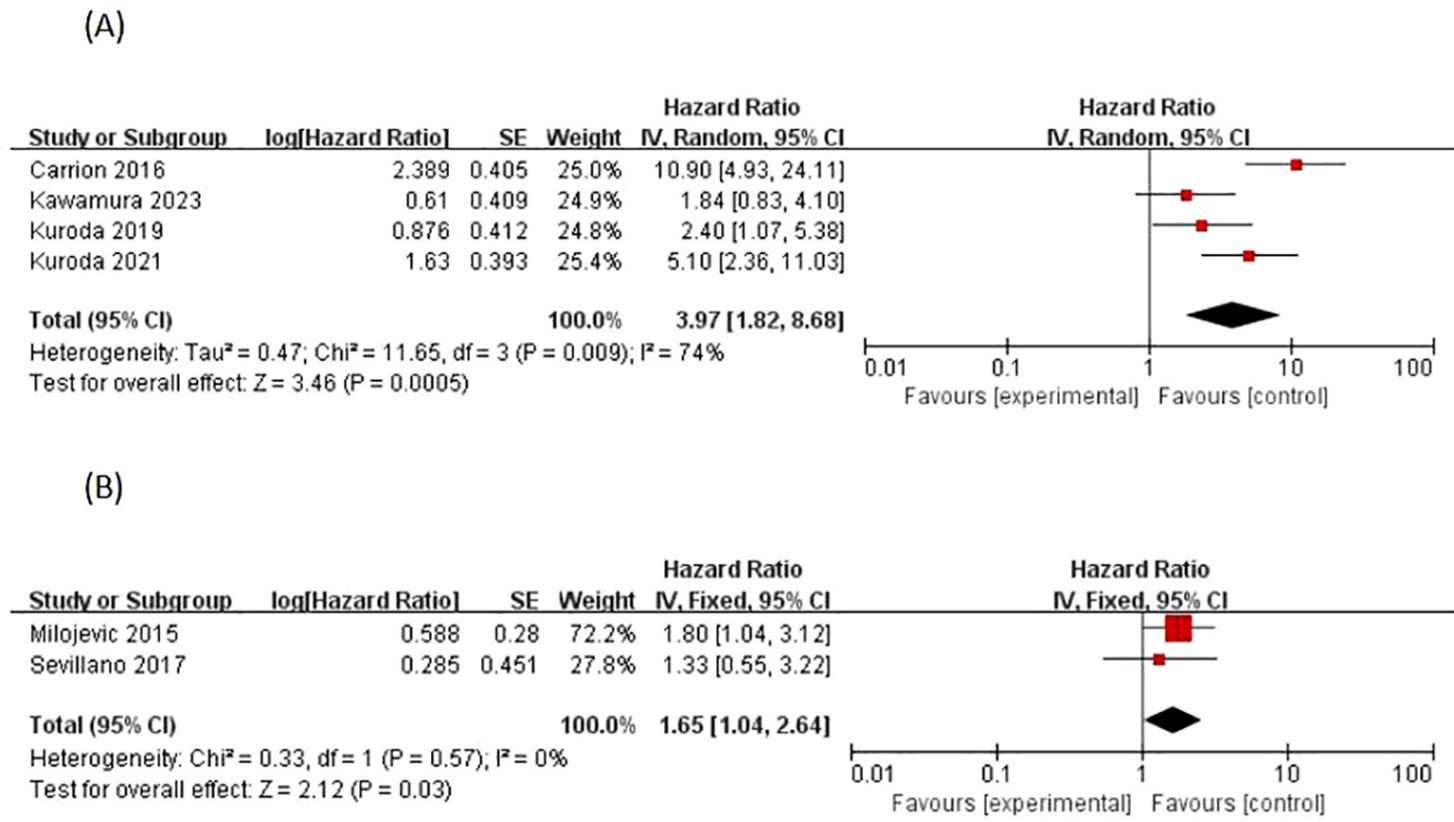
Forrest plots of meta-analyses of Treatment-specific predictors of EUR: **(A)** Positive surgical margins, and **(B)** Adjuvant chemotherapy. EUR, extravesical recurrence.

Four studies (13, 18–20) reported positive surgical margins as a factor for EUR, most of which concluded that there was a significant difference between positive surgical margins and the occurrence of EUR, with Carrion 2016 (13) p < 0.001, Kuroda 2021 (20) p < 0.001, and Kuroda 2019 (19) p = 0.04. The I^2^ statistic was 74% > 50%, and the data Heterogeneity was present, and using a random effects model, a cumulative analysis of available HRs showed that a positive surgical margin (HR 3.97; 95% CI 1.82-8.68; p = 0.0005) was a significant predictor of EUR, as shown in [**Figure 4**].

#### 3.3.2 Adjuvant chemotherapy

Adjuvant chemotherapy regimens as EUR factors were reported in two studies by Milojevic 2015 (23) and Sevillano 2017 (25), with a variability of p = 0.04 < 0.05,p = 0.53 > 0.05, respectively, so there are conflicting results regarding the risk-predictive value of adjuvant chemotherapy and EUR. Homogeneity of outcome data between studies was observed according to the I^2^ statistic of 0% (p = 0.57). A meta-analysis of available HRs showed that adjuvant chemotherapy (HR 1.65, 95% CI 1.04-2.64; p = 0.03) was a significant predictor of EUR, as shown in (**Figure 4**).

Apart from this, in processing the data from the literature, we found that common factors associated with UTUC recurrence, such as smoking history, primary tumor location, and RNU procedure, were not supported by sufficient data and were not shown in our article. It is clear that the exploration of these issues still needs to be followed up vigorously.

## 4 Discussions

Historically, the focus of research on UTUC has predominantly centered on intravesical recurrence following RNU. However, extravesical recurrence, despite its high incidence and the poor prognosis associated with such recurrences and metastases, has received relatively scant attention. Only a limited number of studies have explored extravesical recurrence, and these have failed to converge on a unified perspective regarding the factors that influence urological recurrence post-UTUC. Consequently, no consensus has been reached on this issue. Furthermore, the clinical management of extra-urinary recurrence or metastasis often leaves both clinicians and patients overwhelmed, complicating patient care significantly. Therefore, the current study was initiated to address the aforementioned gaps in research and clinical practice.

Based on a meta-analysis of the available data, significant predictors of EUR were identified, including patient-specific factors [such as proliferating cell nuclear antigen Ki-67, preoperative neutrophil-to-lymphocyte ratio (NLR), and preoperative estimated glomerular filtration rate (eGFR)], tumor-specific factors (such as tumor stage, lymphovascular invasion, and lymph node status), and treatment-specific factors (such as positive surgical margins and adjuvant chemotherapeutic regimens). These predictors should be systematically evaluated to develop a risk-adapted approach for the timely implementation of adjuvant intravesical perfusion chemotherapy and cystoscopic monitoring following RNU. This strategy aims to enhance the safety for urological clinicians and patients with UTUC and to improve patient prognosis quality. We need to emphasize the clinical significance of EUR: Provide important information about patients’ risk of relapse and response to treatment, inform subsequent treatment decisions and patient management, and consider appropriate preventive measures and monitoring strategies in treatment planning.

We observed that tumor stage is strongly associated with extravesical recurrence following UTUC, serving as the most significant predictor in this analysis with a HR of 4.67. This finding aligns with established clinical knowledge, reflecting the general understanding that higher tumor stages correlate with increased risks of metastasis and recurrence. Specifically, for upper urinary tract uroepithelial carcinoma, higher tumor stages and grades are indicative of greater malignancy, deeper tissue infiltration, and a higher propensity for lymphatic and blood channel metastasis (30). Furthermore, tumors with high T-stages, particularly those located in the ureter, are more likely to penetrate the thin ureteral peritoneum, invade the vascular-lymphatic plexus, and thereby increase the likelihood of extravesical recurrence. Previous research supports the view that a pT stage ≥3 is an independent prognostic factor for the progression of EUR, with higher T stages correlating with lower cancer-specific survival. Ouzzane et al. (31) have reported that T stage is a significant risk factor for local recurrence and distant metastasis in UTUC patients who have undergone RNU.

Previous research has demonstrated that a preoperative eGFR below 60 ml/min/1.73 m² is significantly correlated with both disease-specific and recurrence-free survival in patients with UTUC undergoing radical nephroureterectomy. Notably, Ito and Kuroda’s investigations highlighted that ureteral carcinoma, compared to pelvic carcinoma, is more likely to induce obstructive symptoms such as hydronephrosis, increased renal burden, and consequently, a reduction in renal function as indicated by decreased eGFR glomerular clearance (15). Our study also incorporated this preoperative index and confirmed that eGFR is a robust predictor of postoperative EUR in UTUC, with a HR of 3.35. This finding aligns with prior results, suggesting that a lower preoperative eGFR indicates more severe urinary obstruction, greater impairment of renal function, and, given the kidney’s critical role as a metabolic organ, a higher likelihood of recurrence and metastasis in both the internal and external urinary tract.

Our meta-analysis, which included 13 articles, confirmed a strong correlation between Ki-67 expression and EUR in patients with UTUC. Higher levels of Ki-67 were associated with an increased likelihood of EUR. Previous studies have similarly recognized Ki-67 as a significant prognostic marker for UTUC, highlighting its link with EUR. For instance, Jeon et al. reported that overexpression of Ki-67 served as an independent predictor of EUR-free survival (EURFS) and cancer-specific survival (CSS). Additionally, Krabbe et al. conducted a prospective evaluation of Ki-67 in high-grade UTUC patients, further supporting the validity of our findings. Ki-67, a marker of nucleolar proliferation, is widely expressed across various malignant tumors and is considered overexpressed when levels exceed 20% (32–34). Sawazaki et al. (24) explored the relationship between nucleolar phosphoprotein (NPM) and Ki-67, proposing that Ki-67 expression is an independent predictor of EURFS and CSS (35). Their study suggested that Ki-67 might indirectly influence nucleolar function by participating in the phosphorylation process of NPM during mitotic progression and ribosome biogenesis, thereby elucidating the molecular mechanisms linking Ki-67 to EUR.

Our study demonstrated that a higher preoperative NLR is associated with an increased risk of postoperative EUR in patients with UTUC undergoing RNU. This association might be attributed to the role of neutrophils in the inflammatory response, where they not only participate in suppressing anti-tumor immune surveillance but also in remodeling the extracellular matrix (36–39). Conversely, lymphocytes are integral to systemic immune mechanisms; a reduction in their count significantly weakens human immunity, potentially facilitating the metastasis of cancer cells. The European Association of Urology (EAU) guidelines recommend preoperative NLR as a prognostic factor for cancer-specific survival (CSS) in UTUC (40). Furthermore, several studies have reported that inflammatory markers like NLR and platelet-to-lymphocyte ratio (PLR) are linked to poor outcomes in UTUC (41). However, our comprehensive meta-analysis did not find a significant correlation between PLR and EUR occurrence, possibly due to the small sample size included in our study. Additionally, the lack of consensus on defining a critical value for the inflammatory index of PLR may also contribute to these findings. Despite the absence of a definitive positive result for PLR in our analysis, we maintain that both NLR and PLR are crucial for predicting the outcomes in UTUC patients. These markers are easily and conveniently obtained from routine preoperative blood tests, offering potential prognostic value without additional clinical costs.

In our study, lymphovascular invasion (LVI) was identified as a factor strongly associated with EUR after UTUC, aligning with previous research findings. Kuroda’s team demonstrated that elevated fibrinogen levels, positive urocytology, and the presence of hydronephrosis significantly correlate with LVI, suggesting that fibrinogen contributes crucially to the proliferation, bridging, adherence, and metastasis of cancer cells. Furthermore, the presence of positive urocytology and hydronephrosis was consistently linked with LVI (20). Similarly, Sakano et al. confirmed that positive urocytology and hydronephrosis are significantly associated with LVI (42), supporting our results. It is evident from our analysis that LVI is correlated with a higher pathological tumor stage (pT) and an increase in tumor malignancy, thereby elevating the risk of metastasis and recurrence. We also discovered that lymph node metastasis is a positive predictor of EUR. The occurrence of lymph node metastasis significantly increases the likelihood of postoperative EUR in UTUC patients undergoing RNU. Milojevic’s research highlighted a higher incidence of lymph node metastasis (pN⁺) in patients with preoperative anemia (23), suggesting that the aggressive biological potential of tumors in these patients may contribute to their poorer prognosis. Similarly, Luo’s study indicated that patients with positive lymph nodes exhibited a poorer prognosis and a higher incidence of EUR (22), further validating the findings of our study.

Common understanding suggests that metastasis and recurrence are inevitable following positive surgical margins. Our study substantiates this view, revealing that patients undergoing RNU with positive surgical margins exhibited a 3.97-fold increase in the probability of extravesical recurrence compared to those with negative margins. This finding is consistent with previous research, which identifies positive surgical margins as a critical independent predictor of disease-specific death or extravesical recurrence within a shorter timeframe (13, 20). Contrastingly, the 2019 study by Kuroda et al. reported no significant correlation between positive surgical margins and the occurrence of EUR in their dataset (19). Additionally, earlier studies indicated that the recurrence rate of metastasis remained consistent regardless of the laparoscopic technique used for cystectomy, suggesting that recurrence may be more influenced by preoperative factors such as undetected distant metastases rather than surgical technique alone. Furthermore, these findings could be attributed to the limited sample sizes of the studies. Based on our knowledge, the expertise of the urologist plays a crucial role in controlling the extent of tumor resection and minimizing the likelihood of positive margins during surgery.

Our study identified adjuvant chemotherapy as a positive predictor, though not as strong as other factors. Previous studies have shown contradictory and divergent results regarding adjuvant chemotherapy. Ito and Kuroda et al. concluded that neoadjuvant chemotherapy followed by RNU improves the prognosis of patients with combined lymph node metastases of UTUC and that neoadjuvant cisplatin-based chemotherapy prolongs survival (15, 16, 19, 20). However, the study by Dzamic et al. indicates that UTUC is highly sensitive to chemotherapy, but adjuvant cisplatin-based combination chemotherapy is only recommended for patients with pT3 or pT4 or lymph node involvement (14). In some cases, the prognosis of patients treated with adjuvant chemotherapy has not significantly improved. Kawamura’s study observed that adjuvant chemotherapy struggles to eradicate underlying UTUC metastases (17, 18). While this seems counterintuitive, our study found no randomized trials confirming the efficacy of postoperative chemoradiotherapy in UTUC patients. This issue is largely due to the challenges in promoting the use of chemotherapeutic agents in the clinic and in following up on results.

In addition to the positive indicators identified in this manuscript, further specific markers have been observed. Within our clinical practice, it was noted that patients with UTUC who previously had bladder cancer demonstrated an increased risk for EUR. Despite these observations, our study did not show a statistically significant difference regarding the history of bladder cancer, contradicting the findings reported by Luo et al. (21). This discrepancy could be attributed to the limited number of studies and the small sample sizes concerning bladder cancer history included in our analysis, necessitating further investigation by future researchers. Moreover, while Milojevic and Seviliano highlighted the association between anemia and EUR (23, 25), such a correlation was not observed in our dataset. Additionally, several studies have reported a link between positive urinary cytology and recurrence beyond the bladder; however, these findings were not corroborated in our analysis, potentially due to the small sample size and the lack of randomization in the cytological assessments.

This meta-analysis, based on currently available comparative studies, exhibits several notable shortcomings and limitations. Primarily, the analysis included numerous retrospective cohort studies, introducing a considerable degree of heterogeneity. This variation could be attributed to differences in the definitions and measurements of outcomes among the studies. Furthermore, inconsistencies in tumor staging across the studies might have influenced the data analysis; in certain cases, it was not feasible to localize the tumors specifically as renopelvic or ureteral in every patient. Moreover, the sample size for our study was limited, as only a few studies were identified and included through a systematic literature review. Despite these limitations, our study contributes to filling the existing gap in the meta-analysis of prognostic factors linked to postoperative EUR following UTUC.

## 5 Conclusion

Based on the meta-analysis of the available data, we have identified significant predictors of EUR spanning patient-specific (e.g., preoperative Ki-67 levels, eGFR, and NLR), tumor-specific (e.g., tumor stage, lymphovascular invasion, lymph node status), and treatment-specific factors (e.g., positive surgical margins, adjuvant chemotherapy). These findings could propel further investigations into radical renal studies and studies of extravesical recurrence following ureteral resection. Additionally, these insights may provide more practical and applicable decision-making frameworks for the treatment of UTUC patients.

## Data Availability

The raw data supporting the conclusions of this article will be made available by the authors, without undue reservation.
